# Arrhythmogenic cardiomyopathy related *DSG2* mutations affect desmosomal cadherin binding kinetics

**DOI:** 10.1038/s41598-017-13737-x

**Published:** 2017-10-23

**Authors:** Mareike Dieding, Jana Davina Debus, Raimund Kerkhoff, Anna Gaertner-Rommel, Volker Walhorn, Hendrik Milting, Dario Anselmetti

**Affiliations:** 1 Experimental Biophysics and Applied Nanoscience, University of Bielefeld, Bielefeld, Germany; 2 Erich & Hanna Klessmann Institute for Cardiovascular Research and Development, Heart and Diabetes Center NRW, University Hospital of the Ruhr-University Bochum, Bad Oeynhausen, Germany

## Abstract

Cadherins are calcium dependent adhesion proteins that establish the intercellular mechanical contact by bridging the gap to adjacent cells. Desmoglein-2 (Dsg2) is a specific cadherin of the cell-cell contact in cardiac desmosomes. Mutations in the *DSG2*-gene are regarded to cause arrhythmogenic (right ventricular) cardiomyopathy (ARVC) which is a rare but severe heart muscle disease. The molecular pathomechanisms of the vast majority of *DSG2* mutations, however, are unknown. Here, we investigated the homophilic binding of wildtype Dsg2 and two mutations which are associated with ARVC. Using single molecule force spectroscopy and applying Jarzynski’s equality we determined the kinetics and thermodynamics of Dsg2 homophilic binding. Notably, the free energy landscape of Dsg2 dimerization exposes a high activation barrier which is in line with the proposed strand-swapping binding motif. Although the binding motif is not directly affected by the mutations the binding kinetics differ significantly from the wildtype. Furthermore, we applied a dispase based cell dissociation assay using HT1080 cell lines over expressing Dsg2 wildtype and mutants, respectively. Our molecular and cellular results consistently demonstrate that Dsg2 mutations can heavily affect homophilic Dsg2 interactions. Furthermore, the full thermodynamic and kinetic description of Dsg2 dimerization provides a consistent model of the so far discussed homophilic cadherin binding.

## Introduction

**Figure 1:**
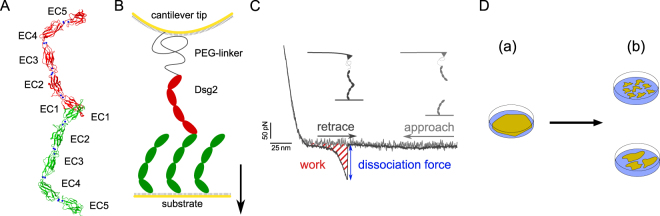
(**A**) Crystal structure of Dsg2 homodimer shown in ribbon representation [^12^, pdbID: 5ERD]. Coordinated Ca^2+^ are shown as blue spheres. (**B**) Schematic of cantilever tip and sample substrate functionalized with Dsg2 for AFM single molecule force spectroscopy. Both, cantilever and substrate are modified with covalently immobilized Dsg2 fragments. (**C**) SMFS working principle and typical force-distance cycle exposing a specific dissociation event. The striated area under the curve represents the dissipated work to break a Dsg2 homodimer. (**D**) Principle of dispase assay for measuring cell-cell adhesion. Cells grown to confluency (a) are lifted up by dispase treatment and exposed to mechanical stress by shaking of cell cultures. Resulting islets of the monolayer are counted and recombinant cell cultures of mutant and wildtype are compared (b).

Cell-cell adhesion is mediated by membrane spanning adhesion proteins that protrude into the intercellular gap and cooperatively build a tight connection along the midline. There are different types of cell junctions which can be distinguished by the type of adhesion proteins and their cellular organization. For instance, adherens junctions comprise classical cadherins that are arranged in loosely ordered structures connecting the actin filamentous network of adjacent cells^1^. In contrast, the intercellular links in desmosomes are established by the cadherin families of desmogleins (Dsg1-4) and desmocollins (Dsc1-3) connecting the intermediate filament network of neighboring cells and revealing a highly-ordered structure^2,3^. Generally, desmosomal and classical cadherin ectodomains comprise of five successively linked conserved cadherin domains (EC1-EC5)^4,5^ (Fig. 1A). The link between each EC domain typically coordinates up to three Ca^2+^ that provide the conformational strain to stretch out the ectodomain^3^. *In vivo* cadherins partially overlap along the midline and dimerize by mutual swapping of domains. This binding motif is driven by exchange of an N-terminal strand in the EC1 domain^6^. A highly conserved tryptophan residue W2 (W51; with regard to specific terminology please refer to Materials and Methods) of the swapping strand can bind into its own hydrophobic pocket to form a closed monomer or it can switch into the corresponding pocket of an opposed EC1 domain to form a swapped strand dimer (Fig. 1A). Due to the two competing binding sites and W2 (W51) residues, respectively, closed monomer and strand swapped dimer states act as competitive inhibitors which are responsible for a low binding affinity^6^. Although, it has been shown that desmosomal cadherins favor a heterophilic type of binding exposing a dissociation constant ${K}_{{\rm{d}}}$ in range of some 10 μm to 100 μm there is also clear evidence for homophilic cadherin dimerization^7–12^.

Dsg2 is a major cadherin of the cardiac desmosome and the only desmoglein expressed in cardiomyocytes. Variances in the *DSG2* gene are associated with severe heart muscle diseases such as ARVC which is characterized by a progressive loss of cardiomyocytes and a fibrofatty tissue replacement predominantly in the right ventricle^13^. In this study, we focussed on the homophilic binding properties of Dsg2 wildtype and two ARVC-related mutations at the single molecule level and in a cell dissociation assay. By means of atomic force microscopy (AFM) based single molecule force spectroscopy (SMFS) we revealed a comprehensive kinetic and thermodynamic description of Dsg2 wildtype and mutant dimerization, respect\ively. Our results confirm a highly specific but low affinity binding which is affected by the mutations. Moreover the activation energy barrier ${\rm{\Delta }}{G}^{\ddagger }$ restricts a fast binding kinetic which is in full accordance with the competitive inhibition of the strand exchange binding motif. Along with the results of the cell dissociation assay both approaches merge into a detailed picture of Dsg2 dimerization kinetics and its influence on cell network stability. These data may contribute to the understanding of *DSG2*-related cardiomyopathy mutations.

## Results

### Dynamic force spectroscopy

Recombinant Dsg2 extracellular cadherin domains 1–4 (EC1-4) were covalently immobilized to AFM-tip and substrate in order to investigate the homophilic binding by means of AFM-SMFS (Fig. 1B). More than 10,000 force distance cycles were recorded at several constant pulling velocities $v$. Dissociation forces $F$ and effective molecular elasticities ${k}_{{\rm{eff}}}$ were extracted from the force distance profiles (Fig. 1C) whose characteristic distributions yield the most probable dissociation force ${F}^{\ast }$ and effective molecular elasticity ${k}_{{\rm{eff}}}^{\ast }$, respectively.

**Figure 2:**
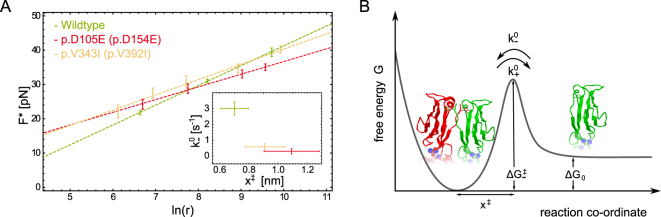
(**A**) Force loading rate graph showing the most probable dissociation force ${F}^{\ast }$ plotted versus the logarithmic loading rate $\mathrm{ln}(r)$. Colors represent Dsg2 wildtype in green, Dsg2- p.D105E (p.D154E) in red and Dsg2-p.V343I (p.V392I) in yellow. The error bars of ${F}^{\ast }$ are estimated by the standard error of the mean (SEM) and were used for a weighted fit of the Evans and Ritchie model (Eq. 2). Inset: The uncertainty of the estimated parameters ${k}_{-}^{0}$ and ${x}^{\ddagger }$ result from the covariance matrix of the fitted model and the uncertainty of the cantilever spring constant. (**B**) Proposed free energy landscape of Dsg2 strand exchange binding exposes a small energy difference between bound and unbound state ${\rm{\Delta }}{G}_{0}$ and a high activation barrier ${\rm{\Delta }}{G}^{\ddagger }$. Dsg2 monomers dimerize when their intermolecular distance go below the reaction length ${x}^{\ddagger }$.

According to the model of Evans and Ritchie we estimated the dissociation rate constant ${k}_{-}^{0}$ and the reaction length ${x}^{\ddagger }$ which is the position of the activation barrier relative to the bound state, by fitting equation 2 to ${F}^{\ast }$ and the corresponding loading rates $r={{k}_{{\rm{eff}}}}^{\ast }\,v$ (Fig. 2A and Table 1). Notably, all experimental series exposed rather small dissociation forces ($< 40\,{\rm{pN}}$) over the full range of pulling speeds (200 nm s^−1^ to 5000 nm s^−1^). Especially at small velocities the dissociation forces can be close to the noise level (approx. 4 pN). Therefore, the experiments demand thorough conduction, a careful data analysis and error handling. Compared to the Dsg2 wildtype which yields a bond lifetime of approx. $\tau =0.33\,{\rm{s}}$ ($\tau ={({k}_{-}^{0})}^{-1}$) the variants p.D105E (p.D154E) and p.V343I (p.V392I) exhibit prolonged bond lifetimes of $\tau =3.39\,{\rm{s}}$ and 1.57 s, respectively. Furthermore, the mutations also reveal extended reaction lengths. Within the estimated error, complex lifetimes and reaction lengths of Dsg2 wildtype and p.D105E (p.D154E) differ significantly. The fitting errors of the binding parameters estimated for p.V343I (p.V392I) overlap at least partially with the error intervals of the two other Dsg2 species (Fig. 2A inset). Moreover, the estimation of ${k}_{-}^{0}$ and ${x}^{\ddagger }$ cannot be done independently. Therefore, the binding properties of p.V343I (p.V392I) apparently differentiate less from Dsg2 wildtype.

**Table 1:** Estimated energy landscape parameters and reaction kinetics of homophilic Dsg2 interactions in equilibrium.

**Dsg2**	${{\boldsymbol{x}}}^{{\boldsymbol{\ddagger }}}$ [nm]	${{\boldsymbol{k}}}_{{\boldsymbol{-}}}^{{\bf{0}}}$[s^−1^]	${\boldsymbol{\tau }}$ [s]	${{\boldsymbol{k}}}_{{\boldsymbol{+}}}^{{\bf{0}}}$[M^−1^s^−1^]	${{\boldsymbol{K}}}_{{\bf{d}}}$ [μM]	${\boldsymbol{\Delta }}{{\boldsymbol{G}}}_{{\bf{0}}}$ [kJ mol^−1^]	${\boldsymbol{\Delta }}{{\boldsymbol{G}}}_{{\boldsymbol{-}}}^{{\boldsymbol{\ddagger }}}$ [kJ mol^−1^]	${\boldsymbol{\Delta }}{{\boldsymbol{G}}}_{{\boldsymbol{+}}}^{{\boldsymbol{\ddagger }}}$ [kJ mol^−1^]
**Wildtype**	0.70(10)	2.99(43)	0.33(5)	7270(1041)	412	18.99(353)	69.05(35)	50.06(318)
**p.D105E (p.D154E)**	1.09(19)	0.30(17)	3.39(190)	361(202)	817	17.32(67)	74.70(136)	57.38(70)
**p.V343I (p.V392I)**	0.91(14)	0.58(20)	1.73(60)	573(198)	1008	16.81(136)	73.06(84)	56.25(51)

Reaction length ${x}^{\ddagger }$, kinetic rate constants of dissociation ${k}_{-}^{0}$ and association ${k}_{+}^{0}$, bond lifetime $\tau$, free energy ${\rm{\Delta }}{G}_{0}$, equilibrium constant of dissociation ${K}_{{\rm{d}}}$, activation energy of dissociation ${\rm{\Delta }}{G}_{-}^{\ddagger }$ and activation energy of association ${\rm{\Delta }}{G}_{+}^{\ddagger }$.

**Figure 3:**
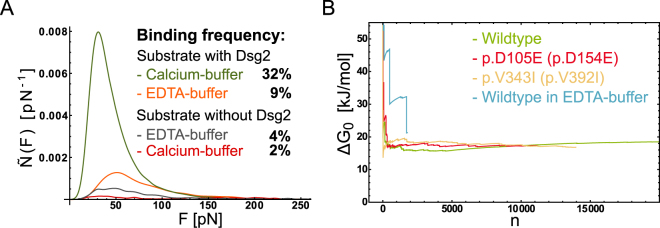
(**A**) Series of control experiments for different Ca^2+^ concentrations and sample substrates. The dissociation probability distributions are scaled for the absolute binding frequency. Experiments with Dsg2 modified AFM cantilever and sample substrate in 2 mM calcium-buffer (green) and EDTA-buffer (orange) expose a clear dependency of the binding frequency on the calcium concentration. The background of unspecific adhesion was tested in an experiment conducted on substrates lacking Dsg2 in calcium (red) and EDTA-buffer (gray). (**B**) Evolution of the Jarzynski equality. The exponentially averaged work ${W}_{n}$ plotted versus the number of dissociation events $n$. For wildtype Dsg2 and mutations thereof (green, red and yellow) measured in a 2 mM Ca^2+^ buffer-solution the Jarzynski equality converges fast yielding a robust estimate of the free energy difference ${\rm{\Delta }}{G}_{0}$. The evolution of ${\rm{\Delta }}{G}_{0}$ for wildtype Dsg2 (blue) in calcium-free EDTA-buffer solution does not converge for $n < 2000$.

In order to prove the specificity of the homophilic Dsg2 interaction and to rule out unspecific adhesion we conducted a series of control experiments. AFM force distance cycles were recorded in calcium-buffer and calcium-free EDTA-buffer as well as on gold substrates lacking Dsg2. Our data revealed that Dsg2 dimerization critically depends on the calcium concentration (Fig. 3A). We observed a binding frequency of 32% at a Ca^2+^ concentration of 2 mM, whereas in EDTA-buffer the binding probability dropped to 9%. Furthermore, the dissociation force probability distributions of experiments conducted in EDTA-buffer yield a broad shape and multiple shallow peaks that can be attributed to multifold unspecific adhesion events. Furthermore, the binding frequency on substrates lacking Dsg2 drops significantly to 4% (EDTA-buffer) and 2% (calcium buffer) proving that the background of unspecific adhesion on substrates lacking Dsg2 was minute and therefore does not affect the results.

### Reconstruction of energy landscapes

Jarzynski’s equality (Eq. 3) allows to relate mechanical work $W$ measured under non-equilibrium conditions with the free energy difference ${\rm{\Delta }}{G}_{0}$ in thermodynamic equilibrium^14^. Here, ${\rm{\Delta }}{G}_{0}$ represents the free energy difference between the bound and unbound state of Dsg2^15^. We determined the work ${W}_{{\rm{n}}}$ that was dissipated to break n individual Dsg2 dimers by calculating the area under the corresponding force curves (Fig. 1C). Subsequently, the data set ${W}_{{\rm{n}}}$ was averaged exponentially using Jarzynski’s equality. This term converges rapidly for all Dsg2 species providing a robust estimate of ${\rm{\Delta }}{G}_{0}$ (Fig. 3B). Notably, Dsg2 wildtype and mutants yield almost identical binding energies of approx. 18(1) kJmol^−1^ (Table 1) coinciding with a preserved binding motif. Moreover, we used Arrhenius’ equation (Eq. 5) to estimate the activation energy ${\rm{\Delta }}{G}_{+/-}^{\ddagger }$ of the association and dissociation path, respectively. Dsg2 wildtype revealed ${\rm{\Delta }}{G}_{-}^{\ddagger }\approx 69$ kJ mol^−1^ whereas the data for the Dsg2 mutants p.D105E (p.D154E) and p.V343I (p.V392I) yielded a slightly higher energy of approx. 75 kJ mol^−1^ and 73 kJ mol^−1^, respectively (Table 1). The equilibrium constant of dissociation ${K}_{{\rm{d}}}$ was estimated as 412 μM for the wildtype Dsg2 which proves the expected low affinity of Dsg2 dimerization. The mutants p.D105E (p.D154E) and p.V343I (p.V392I) yield an even smaller affinity of 817 kJ mol^−1^ and 1008 kJ mol^−1^, respectively. Furthermore, the association rate constants ${k}_{+}^{0}$ were estimated according to equation 4 (Table 1). In total, an approximation of the free energy landscape (Fig. 2B) together with the binding kinetics provide a detailed picture of the homophilic interaction of Dsg2.

### Cell-cell adhesion assay

**Figure 4:**
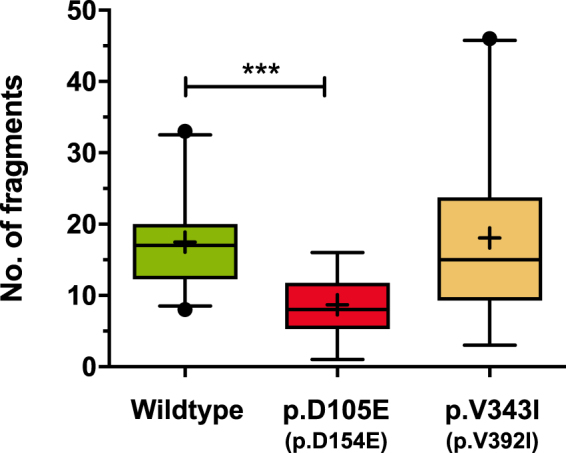
Confluent monolayers of transfected cells were subjected to dispase assay. Decreased number of fragments indicates relatively enhanced cell-cell adhesion. Box and whiskers plots represent the number of fragments in six independent experiments for Dsg2 wildtype, p.D105E (p.D154E; $p < 0.001$ versus wildtype) and p.V343I (p.V392I). Box and whiskers plot: boxes extend from the 25th–75th, whiskers from the 5th–95th percentiles, the medians are shown as lines; outliers are given as dots. Means are indicated as crosses.

In parallel we investigated the Dsg2 interactions at the cellular level by a dispase-based cell dissociation assay using recombinant human HT1080 cells (Fig. 1D). The Dsg2-wildtype delivered on average 17.5(63) monolayer fragments, whereas the mutant Dsg2 p.D105E (p.D154E) could be fragmented into less pieces (8.7(43); $p < 0.001$) of the monolayer, suggesting a stronger cellular interaction. Of note, the number of fragments in the dispase assay of the mutant p.V343I (p.V392I) was not significantly different from wildtype Dsg2 (18.0(123); Fig. 4).

## Discussion

In this study we revealed a detailed picture of homophilic Dsg2 interaction kinetics and thermodynamics of the Dsg2 wildtype and arrhythmogenic cardiomyopathy related mutant protein. By means of AFM-SMFS we estimated the binding properties of Dsg2. The W2 (W51) residue and the associated hydrophobic pocket which are critical for strand swapping are highly conserved throughout desmosomal and classical cadherins^16^. Therefore, kinetic and thermodynamic binding parameters estimated for Dsg2 are expected in the range of other strand swapping cadherins. For the Dsg2 wildtype homo-complexes we found an average lifetime of 0.33s. Similarly, recent reports on the dimerization of Dsg1 and C-cadherins found complex lifetimes of 0.17s and 0.91s, respectively^9,11^. Moreover, the equilibrium constant of dissociation was estimated as ${K}_{{\rm{d}}}=412$ μM which is in perfect agreement with ${K}_{{\rm{d}}}=433$ μM obtained by Harrison and co-workers in an sedimentation equilibrium analytical ultracentrifugation assay^12^. In a solution NMR approach E-cadherins exhibited an equilibrium dissociation constant of ${K}_{{\rm{d}}}=720$ μM^17^.

Notably, Dsg2 association is apparently inhibited what can be seen by the association rate constant of ${k}_{+}^{0}=7270$M^−1^s^−1^ which is orders of magnitude smaller than a diffusion limited association^18,19^. Accordingly, we could determine a height of the activation energy barrier of association (${\rm{\Delta }}{G}_{+}^{\ddagger }=50$ kJ mol^−1^, eqs 5 and 6) which is significantly larger than the difference in free energy ${\rm{\Delta }}{G}_{0}$ (Table 1). This constraint binding reaction is most likely due to the competitive inhibition of the N-terminal strand exchange^6^. Our experiments furthermore demonstrate that the Dsg2 dimerization probability critically depends on the Ca^2+^ concentration which is in full agreement with data published elsewhere^8,9,11,20–23^ (Fig. 3A). Like classical cadherins, Dsg2 comprises three calcium coordination sites between each EC domain except for the linker region between EC3 and EC4 which comprises only two binding sites^12^. Interdomain calcium binding straighten the Dsg2 ectodomains to their physiological conformation which is prerequisite to span the intercellular gap^3^. The incomplete calcium coordination between EC3 and EC4 however, might be responsible for the sharp bend found in crystal structures^12^. Moreover, calcium coordination between EC1 and EC2 provides conformational strain that releases the N-terminal W2 (W51) residue from its hydrophobic binding pocket and increases its diffusional mobility. This transformation to an open momomer is prerequisite for mutual strand exchange between Dsg2 monomers^24,25^. Still, dissociation force probability distributions obtained in Ca^2+^ -free EDTA-buffer support these findings. The binding frequency drops and the force distribution exposes a rather broad shape and multiple shallow peaks which suggests that in absence of Ca^2+^ Dsg2 dimerizes in an unspecific manner. Finally, the Jarzynski sum of exponentially weighted adhesion energies ${W}_{n}$ does not converge which also can be attributed to an unspecific interaction within EDTA-buffer solution (3B).

In a comparative analysis of Dsg2 wildtype and variants we generally observed prolonged bond lifetimes and increased activation barriers for the Dsg2 mutants (Table 1), which results in a significantly reduced affinity of ${K}_{D}=817$ μM for p.D105E (p.D154E) and ${K}_{D}=1008$ μM for p.V343I (p.V392I). Consequently, the association rate is dramatically reduced for both variations p.D105E (p.D154E) (${k}_{+}^{0}=361$ M^−1^s^−1^) and p.V343I (p.V392I) (${k}_{+}^{0}=573$ M^−1^s^−1^), respectively. Even though Dsg2 affinity and kinetics are affected by the mutations, the binding free energy is comparable to the wildtype coinciding with a preserved strand swapping binding motif. Interestingly, Dsg2 p.D105E (p.D154E) bears a mutation at a Ca^2+^ binding site within the first interdomain linker region^3^. Recently published molecular dynamics simulations for E-cadherins revealed that calcium binding between EC1 and EC2 increases the mobility of the N-terminal strand supporting dimerization by strand swapping^24^. Therefore, we assume that an impaired Ca^2+^ coordination of Dsg2 p.D105E (p.D154E) leads to a deficient release or mobility of the N-terminal strand. As a consequence the strand exchange binding is evidently inhibited, which slows down Dsg2 dimerization. We tested in parallel the cellular effects of the *DSG2* mutations by a more complex dispase assay. This assay is a measure for the strength of the cellular interaction, which is based on the dissociation of a cell culture monolayer. The number of fragments resulting from the dispase assay was significantly lower when transfected with the cDNA for Dsg2 p.D105E (p.D154E), but not for p.V343I (p.V392I) in comparison to wildtype Dsg2. Thus, these experiments suggest that the resulting cell-cell contacts were indeed stronger for the mutant Dsg2 p.D105E (p.D154E), which is in agreement with the AFM-SMFS data on the highly increased lifetime of this mutant ($\tau =3.39$ s) in comparison to the wildtype ($\tau =0.33$ s). Nevertheless, due to the retarded association ${k}_{+}^{0}$ of Dsg2 p.D105E (p.D154E), these homocomplexes expose an increased equilibrium constant of dissociation ${K}_{{\rm{d}}}$ (Table 1) indicating a smaller number of intact bonds at a time. This result evidently disagrees with the increased adhesion observed for p.D105E (p.D154E) cellular monolayers. However, this at the first sight contradictory behavior can be consistently explained when regarding the conditions within the cell-cell contact. Desmosomes are highly ordered adhesive interfaces with a cadherin density of 17,500 *μ*m^−2^ in case of epidermal desmosomes leaving a single cadherin a cell surface area of less than $8\times 8$ nm^2^ ^2,3^. This compact arrangement evidently leads to a spatial confinement within the desmosome which constrains diffusion of unbound cadherin ectodomains. Moreover, Dsg2 p.D105E (p.D154E) exhibits the longest reaction length of all tested Dsg2 variants (${x}^{\ddagger }\approx 1.1\,{\rm{nm}}$; Table 1) which can be interpreted as a larger reach of the potential molecular interaction promoting fast (re-) association. Opposing Dsg2 p.D105E (p.D154E) monomers can therefore effectively bind at larger intermolecular distances (Fig. 2B) resulting in an apparently enhanced association rate. As a consequence, association rate constants estimated in solution or single molecule assays do not apply for membrane bound cadherins within dense desmosomes. Instead, the spatial restriction and an increased interaction length most likely force fast (re-) association of Dsg2 p.D105E (p.D154E) homocomplexes and an increased cell network stability. However, although we also identified aberrant binding parameters for the mutation p.V343I (p.V392I) ($\tau =1.73$ s) we could not find a significant difference from the wildtype Dsg2 in the cellular assay. Of note, the dispase assay is not restricted to the homophilic interaction of Dsg2, but also reflects potential interactions with other cell-cell contact proteins like i.e. the desmosomal cadherin desmocollin 2, which is also a binding partner of Dsg2 and coexpressed in HT1080 cells. As a consequence, the homophilic molecular interaction determined by AFM-SMFS reflects only one potential molecular interaction within the desmosome. Thus, although we found a difference for the binding parameters of this mutant, these molecular properties of p.V343I (p.V392I) do not converge in the disturbance of the cellular interaction *in vitro*. Both mutations are listed as variants of unknown significance (VUS; http://www.arvcdatabase.info), but solid calculations of the disease penetrance and differences in the clinical phenotypes are not available. Data from our study do not support a pathogenic dominant role of the mutation p.V343I (p.V392I). We speculate that our *in vitro* data on the homophilic interaction might indicate that this particular mutation may be a modifier *in vivo*. However, these *in vitro* effects for the homophilic Dsg2 and cell-cell interaction in this study might be not directly transferrable to the *in vivo* situation and should therefore be carefully interpreted in the clinical context. In summary, the mutant Dsg2 analyzed *in vitro* do not result in a weaker binding energy, but in an increased binding lifetime and aberrant association and dissociation parameters. This indicates that especially the protein integration and degradation of the desmosomal cadherins in the desmosome might be affected in cardiomyopathy related *DSG2* mutations.

## Methods

### Nomenclature of aminoacids and mutations

Medical and biophysical publications commonly use disparate nomenclature of aminoacids and mutations. In biophysical context it is good practice to identify specific aminoacids based on the position in the mature protein whereas in medical publications and databases they are addressed on the basis of the open reading frame (ORF). In order to avoid confusion and for a comprehensive reading of this paper we used the nomenclature of the aminoacid position based on the position in the mature protein and in parallel the numbering within the ORF which is given in brackets.

### Recombinant expression of Dsg2

The extracellular regions EC1-EC4 of Dsg2 were stably transfected in HT1080 cells as previously reported^26^. Expressed Dsg2 was purified from the cell culture supernatant by affinity chromatography on HisTrap excel columns (GE Healthcare, Solingen, Germany), according to the manufacturer’s instructions. The protein solutions were concentrated with Amicon Ultra-15 Centrifugal Filter Units (10,000 MWCO, MerckMillipore, Darmstadt, Germany). Proteins were identified by Western blotting and analyzed by means of SDS-Page with Coomassie-brilliant-blue staining, revealing a purity of at least 90%. The concentration of Dsg2 varied between 0.8 gL^−1^ 1.5 gL^−1^.

### Functionalization of AFM tips and substrates

Glas substrates were metallized by physical vapor deposition (PVD) with a 3 nm layer of chrome followed by 120 nm gold using a commercially available PVD system (MED020 Coating System, Leica Microsystems, Wetzlar, Germany). Soft gold coated silicon nitride cantilevers (BL-RC150VB, Olympus, Tokio, Japan) with a nominal spring constant of 30 pNnm^−1^ were cleaned with acetone and ethanol. Both, metallized glass substrates and cantilevers were coated with a self-assembled monolayer (SAM), incubated in an ethanolic solution of 1 mM 6-amino-1-hexanethiol-HCl (Sigma-Aldrich, Saint Louis, MO, USA) for 1.5 h and washed subsequently with ethanol. Cantilevers and substrates were incubated in a 100 μM water-free dimethyl sulfoxide (DMSO) solution of different hetero-bifunctional polyethylene glycol (PEG) cross-linkers for 1.5 hours at room temperature. The force probes were functionalized with the maleimide-PEG-succinimidyl valerate 3,400 cross-linker (Laysan Bio Inc., Arab, AL, USA), whereas the gold coated glass substrates were modified with NHS 3-maleimideopropionate (Celares, Berlin, Germany). Notably, both cross-liners bear identical functional groups. However, the linker used to couple Dsg2 to the cantilevers is significantly longer (approx. $25\,{\rm{nm}}$). Apart from providing a covalent link to the cantilever the linker adds steric elasticity to the molecular system and it ensures that dissociation events occur far from the surface. Both is required to align the force extension curves and to discriminate between specific and unspecific binding events. Finally, AFM probes and substrates were incubated in 20 μL Dsg2 solution without further dilution at 5 °C until measurement. The free maleimide of the cross-linkers binds to the C-terminal thiol side chain of a unique cystein C447 (C496) within the recombinant Dsg2 fragment. Both, cantilevers and substrates could be used without loss of activity for up to four days.

### DFS experiments

SMFS was performed with a commercially available Multimode 8 AFM system (Bruker, Santa Barbara, CA, USA). Before each experimental series the cantilevers were calibrated by the thermal fluctuation method^27^. All force measurements were conducted in calcium and EDTA buffer, respectively (2 mM CaCl2, 10 mM HEPES, 150 mM NaCl, pH 7.4 and 5 mM EDTA, 1 mM EGTA, 10 mM HEPES, 150 mM NaCl, pH 7.4). Prior to buffer changes substrates and cantilevers were extensively rinsed with washing buffer (10 mM HEPES, 150 mM NaCl, pH 7.4). Up to 20,000 force distance cycles were performed for each pulling velocity ranging from 200 nm s^−1^ to 5,000 nm s^−1^. The characteristic non-linear force ramps were individually analyzed within the theoretical framework of the worm like chain model (WLC) to rule out multiple saw tooth-shaped rupture events. Furthermore, rupture events with a dissociation length larger than 60 nm were discarded as the whole molecular complex (linkers and Dsg2 dimer) exhibits a length of approx. 60 nm. Detected dissociation forces were accepted for analysis when they exceeded the noise level (here approx. 4 pN) by a factor of three. The most probable dissociation force ${F}^{\ast }$ was determined for each pulling velocity using a kernel density estimation (KDE)^28,29^. The corresponding probability density function ${\tilde{P}}_{n}(F)$ was determined as:

1 $${\mathop{P}\limits^{ \sim }}_{n}(F)=\frac{1}{n\,\sqrt{2\pi }}\sum _{i=1}^{n}\frac{1}{{F}_{{\rm{R}}{\rm{M}}{\rm{S}}i}}\exp (-\frac{{(F-{F}_{i})}^{2}}{2\,{F}_{{\rm{R}}{\rm{M}}{\rm{S}}i}^{2}}).$$

Here, ${F}_{i}$ and ${F}_{{\rm{RMS}}i}$ denote the i-th element in a 1 by $n$ array of dissociation forces and the root mean square (RMS) noise of the force, respectively. The most probable molecular elasticity was calculated likewise and multiplied by the corresponding pulling velocity to yield the effective loading rate $r={{k}_{{\rm{eff}}}}^{\ast }\,v$. In order to estimate the dissociation rate constant in thermodynamic equilibrium under zero force ${k}_{-}^{0}$ and the reaction length ${x}^{\ddagger }$ we applied the model of Evans and Ritchie^30^:

2 $${F}^{\ast }=\frac{{k}_{{\rm{B}}}\,T}{{x}^{\ddagger }}\,{\rm{l}}{\rm{n}}(\frac{{x}^{\ddagger }\,r}{{k}_{-}^{0}\,{k}_{{\rm{B}}}\,T}).$$

### Estimate free energy difference using Jarzynski’s equality

When estimating thermodynamic binding parameters such as the free energy difference ${\rm{\Delta }}{G}_{0}$ between bound and unbound state or the dissociation constant ${K}_{{\rm{d}}}$ from AFM-SMFS data it is good practice to assume a diffusion limited association rate constant ${k}_{+}^{0}$ ^31–33^. However, this means does not apply for systems in which free diffusion is possibly restricted. Instead, Jarzyski’s non-equilibrium work theorem allows calculating ${\rm{\Delta }}{G}_{0}$ directly from a series of non-equlibrium experiments no matter how far from equilibrium the system was probed^14,15^. To estimate ${\rm{\Delta }}{G}_{0}$ we used Jarzynski’s equality:

3 $$\exp (-\frac{{\rm{\Delta }}{G}_{0}}{{k}_{{\rm{B}}}\,T})={\langle \exp (\frac{{W}_{n}}{{k}_{{\rm{B}}}T})\rangle }_{n}.$$

Here, ${W}_{n}$ is the work that is dissipated to break an individual bond (${W}_{n} < 0$). A sufficiently large set of work measurements ($n\to \infty$) obtained far from equilibrium, exponentially averaged, converge towards the free energy difference in thermodynamic equilibrium ${\rm{\Delta }}{G}_{0}$ and therefore give an accurate estimate. We used a custom-made MATLAB software to validate and analyze the characteristic nonlinear force ramps that mainly result from the stretching of the PEG cross-linker. In brief, the force profiles are corrected for the molecular extension and possible position and deflection offsets. A worm-like chain fit is applied to the data yielding the contour length of the molecular complex. We estimated the overall length of a Dsg2 dimer ($31.5\,{\rm{nm}}$) from crystal structure experiments whereas the length of the PEG cross-linker is well-known (approx. $25\,{\rm{nm}}$) [^12,34^, pdbID: 5ERD]. Filtering the dataset for a distinct contour length (here, $56.5\,{\rm{nm}}$) results in a set of well-aligned force profiles. Subsequently, we estimated a set of ${W}_{n}$ by determining the area under the force profiles^14,15^. Finally, ${W}_{n}$ is weighed by applying the Jarzynski equality and solved for ${\rm{\Delta }}{G}^{0}$. The equilibrium constant ${K}_{{\rm{d}}}$ and the association rate constant ${k}_{+}^{0}$ in thermodynamic equilibrium are calculaded as:

4 $${K}_{{\rm{d}}}=\exp (-\frac{{\rm{\Delta }}{G}_{0}}{{N}_{{\rm{A}}}\,{k}_{{\rm{B}}}\,T})=\frac{{k}_{-}^{0}}{{k}_{+}^{0}}.$$

In order to estimate the activation energy of the dissociation path ${\rm{\Delta }}{G}_{-}^{\ddagger }$ we used the Arrhenius equation^35–37^:

5 $${k}_{-}^{0}(T)=\nu \,\exp (-\frac{{\rm{\Delta }}{G}_{-}^{\ddagger }}{{N}_{{\rm{A}}}\,{k}_{{\rm{B}}}\,T}).$$

where ${N}_{{\rm{A}}}$ is the Avogadro constant and $\nu =\tfrac{{k}_{B}T}{h}=6\times {10}^{-12}{{\rm{s}}}^{-1}$ is the attempt frequency for the crossing of the transition state. Accordingly, the activation energy of association is estimated as:

6 $${\rm{\Delta }}{G}_{+}^{\ddagger }={\rm{\Delta }}{G}_{-}^{\ddagger }-{\rm{\Delta }}{G}_{0}\mathrm{.}$$

### Cell–cell adhesion assay

Cell–cell adhesion was investigated using a dispase-based cell dissociation assay^38^. Stably transfected full-length-*DSG2* (wildtype and mutants p.D105E (p.D154E) and p.V343I (p.V392I))-pEYFP/DSC2b-pLPCX HT1080 cells were seeded in 12-well plates and grown to confluence in the presence of 2 mM CaCl_2_. After two washes with phosphate-buffered saline, the adherent cells were incubated at 37 °C for 20 minutes with 0.3 ml dispase II (2.4 U/ml, Sigma Aldrich, Darmstadt, Germany) resulting in a non-adherent cell monolayer. Released monolayers were rotated on an orbital shaker (150 rpm) for 20 minutes. Finally, cell dissociation was calculated by counting monolayer fragments. Every experimental condition was performed in quadruplicate and repeated five times. Values are given as means $\pm$ SD; statistical analysis was performed with one-way ANOVA and Dunnett’s Multiple Comparison Test using GraphPad Prism (GraphPad Software, La Jolla, CA, USA).

## Conclusion

Using AFM-SMFS we estimated the dimerization kinetics and thermodynamics of Dsg2 wildtype and two mutations. Furthermore, we tested the impact of these mutations on the stability of cellular networks by means of a dispase based cell-cell adhesion assay. In combination, our results provide a fascinating glimpse into the complex molecular interplay within the cardiac desmosome. Briefly, we found that Dsg2 wildtype and both tested mutations differed with respect to their binding kinetics. Dsg2 wildtype exhibited the smallest complex lifetime ($\tau =0.33\,{\rm{s}}$) whereas p.D105E (p.D154E) and p.V343I (p.V392I) exhibited considerably increased lifetimes of $\tau =3.39\,{\rm{s}}$ and $\tau =1.73\,{\rm{s}}$, respectively. Estimating the difference in free energy ${\rm{\Delta }}{G}_{0}$ by means of Jarzynski’s equality allowed us to estimate the equilibrium constant of dissociation ${K}_{\text{d}}$ and the association rate ${k}_{+}^{0}$. Interestingly, Dsg2 wildtype homodimers exhibited the highest affinity (${K}_{{\rm{d}}}=412\,\mu {\rm{M}}$) among the analyzed Dsg2 species. As a consequence the association rate of Dsg2 wildtype is one order of magnitude higher compared to both mutations. Despite the fact that Dsg2 wildtype exhibits the highest affinity cellular networks bearing the very same cadherin should expose higher resistance against tensile stress than p.D105E (p.D154E) and p.V343I (p.V392I). Nevertheless, cell cluster stability was increased for p.D105E (p.D154E) which contradicts to our single molecule results.Taking into account spatial restrictions within the dense highly ordered desmosome, we can conclude, that due to limited diffusion unbound cadherin ectodomains can (re-) associate considerably faster leading to an increased cluster stability as observed for p.D105E (p.D154E). The stability of Dsg2 homocomplexes within the desmosome is apparently governed by their bond life times as varying association rates in solution are compensated by confined diffusion and dense configuration within the intercellular gap. Evidently, the integrity of the cardiac muscle is based on the weak but cooperative binding between cadherin ectodomains. The fast binding kinetics of Dsg2 wildtype raises the question of the physiological reason. Our results imply that the cardiac desmosome can be regarded as a tight but highly dynamic connection between individual heart muscle cells. This well-balanced molecular interaction suggests that the cardiac desmosome is able to continuously tune its configuration to the alternating contractions of the heart muscle.
